# Cellular and molecular basis associated with metacestode proliferation in subarachnoid neurocysticercosis

**DOI:** 10.3389/fimmu.2022.1025599

**Published:** 2022-10-10

**Authors:** Miguel A. Orrego, Manuela R. Verastegui, Hector H. Garcia

**Affiliations:** ^1^ Laboratory of Immunopathology in Neurocysticercosis, Facultad de Ciencias y Filosofía, Universidad Peruana Cayetano Heredia, Lima, Peru; ^2^ Infectious Diseases Research Laboratory, Facultad de Ciencias y Filosofía, Universidad Peruana Cayetano Heredia, Lima, Peru; ^3^ Center for Global Health, Universidad Peruana Cayetano Heredia, Lima, Peru; ^4^ Cysticercosis Unit, Instituto Nacional de Ciencias Neurológicas, Lima, Peru

**Keywords:** *Taenia solium*, neurocysticercosis, subarachnoid cyst, proliferation, germinative cells

## Introduction

*Taenia solium* is a cestode parasite with a complex life cycle that includes two mammalian hosts: pigs and humans (1, 2). Humans are the only definitive host, in whose small intestine develops and lives the adult form of the parasite; this infection is known as taeniasis and makes humans the most important reservoir and disseminator of the parasite.

Pigs act as intermediate hosts by carrying the larval stages or cysts (porcine cysticercosis) (3). Pigs become infected after consuming feces contaminated with the eggs, and cysts commonly develop in muscles or subcutaneous tissue (3). Humans can also act as accidental intermediate hosts and develop cysticercosis by ingestion of *T. solium* eggs (2, 3).

Infection of the human central nervous system (CNS) with the cysts of the parasites is known as neurocysticercosis (NCC) and is the most important cause of acquired epilepsy worldwide (4). NCC patients present very heterogeneous symptoms and the severity of these manifestations are associated with the parasite burden, anatomical location of the lesions, degree of parasite degeneration, the host immune response, and parasite development (4, 5).

In human NCC, the cysts are localized mainly in the brain parenchyma and remain isolated and protected from the host immune system by the blood-brain barrier (BBB) (6). In addition, the cyst establishes mechanisms for evasion of host immune response (7). This host-parasite equilibrium is vital for the survival of the parasites for many years. The presence of immunoglobulins (IgM, IgG, IgA, and IgE) on the parasite surface has been reported, suggesting cyst masking with the host’s antibodies through the Fc receptor present in the tegument of the cysts (8). Other mechanisms reported to evade the immune response are the inhibition of the complement cascade or lymphocyte proliferation through the secretion of the paramyosin and the metacestode factor (MF), respectively (9, 10).

Analysis of MF revealed that it is a small RNA molecule, but its full characterization has not yet been possible (11). However, in the last decade, small non-coding RNAs, such as microRNAs, have emerged as important factors in diverse biological processes (12). MicroRNAs have a significant role in the control of gene expression, and alterations in the expression levels of some host microRNAs are associated with seizures and epilepsy (13). It is not surprising to consider that some microRNAs would have active participation during NCC (14). The identification of microRNAs secreted by the parasite allows us to understand new pathways of action on the host; they also constitute potential markers for the diagnosis of the disease and the follow-up of patients after the initiation of anthelmintic treatment.

After a prolonged period of years or even decades (15), the parasite degenerates naturally or by the use of anthelmintic drugs, releasing antigens that trigger an inflammatory reaction. The cysts are surrounded by a dense fibrous layer rich in collagen, epithelioid cells, multinucleated giant cells, and abundant immune cells such as CD8^+^ T lymphocytes, plasma cells, and in smaller proportion CD4^+^ T lymphocytes. The fibrotic capsule contains abundant new vessels or capillaries suggesting angiogenesis processes. Additionally, eosinophils are observed with activation and proliferation of microglia, astrogliosis and subsequent glial scar formation, edema, neuronal degeneration, and a strong peripheral response characterized by a mixed profile of Th1/Th2 cytokines (15, 16). The precise mechanisms underlying the characteristics and dynamics of the Th1 and Th2 immune response still need to be elucidated.

Subarachnoid NCC is the less common but more severe form of extraparenchymal NCC, occurring when the cyst develops in the basal subarachnoid region or Sylvian fissure of the brain (17). Subarachnoid NCC is characterized by the presence of an aberrant and proliferative form of the parasite (racemose cyst), reaching several centimeters in diameter, composed of multiple interconnected vesicles (18). Subarachnoid cysts frequently lack the scolex, resulting in an abnormal and sterile form of the parasite (17, 18).

The expansion of subarachnoid NCC within CNS is associated with a combination of mass-effect and inflammatory pathologies such as displacement of the surrounding nervous tissue, intense inflammatory reaction, fibrosis, thickening of the leptomeninges, hydrocephalus, intracranial hypertension, and meningitis; associated with significant mortality (20%) in most reported cases (17, 18).

The treatment of subarachnoid NCC requires the administration of anthelmintic drugs in combination with anti-inflammatory drugs, commonly corticosteroids, in many cases for long periods of time to control the inflammatory response and parasitic infection (19) and frequently also requires surgical intervention.

The onset of symptoms in subarachnoid NCC occurs after very long incubation periods (1.5-2 decades) (19), time required for the development and expansion of the cyst. There are many biological and histological differences between subarachnoid cysts and parenchymal cysts. The most important is the exacerbated ability of subarachnoid cysts to proliferate. Histological observations demonstrate that the bladder wall of the subarachnoid cyst presents a disordered arrangement that differs in the quantity of the main tissue components (20). These features suggest a tumor-like proliferation that preserves the histological characteristics of the parasite but with an abnormal organization. Some authors have postulated that the degeneration of the scolex is associated with the proliferation of the bladder wall in the subarachnoid cysts.

The events that promote the transformation of a quiescent nonproliferative form such as the parenchymal cyst to the proliferative sterile subarachnoid cyst are poorly understood; however, we have recently demonstrated the presence of a particular group of stem cells (germinative cells) throughout the bladder wall of the subarachnoid cyst (21). These cells constitute mitotically active cells that, in parenchymal cysts, are limited to the base of the neck for the development of tapeworm after the cysts are ingested by a suitable intermediate host (22) and would be responsible for the continuous proliferation and expansion of the bladder wall of the subarachnoid cyst. These findings suggest that the presence of germinative cells in the bladder wall of the subarachnoid cyst would result from the incorporation or assimilation of the scolex during the early stages of development of the subarachnoid cyst.

The natural development of the cyst involves close interactions with the host cells; these interactions allow the normal development of the parasite and regulate its homeostasis and cellular processes (7). One of the main mechanisms of cell-cell communication is through biomolecules such as hormones or cytokines. Among the main signaling pathways identified in cestodes are insulin, transforming growth factor-β (TGF-β), epidermal growth factor (EGF) and wingless/integrated protein (WNT) (23–27). Although tyrosine or serine/threonine kinase-type surface receptors and downstream mediators of these pathways have been identified in proliferative species such as *Echinococcus multilocularis* or *Taenia crassiceps*, their involvement in the development and continued growth of the subarachnoid cyst has been poorly studied.

We have also evaluated the action of host hormones on subarachnoid cysts growth employing primary cultures of germinative cells isolated from the bladder wall and revealed the sensitivity of this cell population to the action of insulin by promoting its proliferation (21). Additional assays evaluated the downstream signaling pathway of insulin through mitogen-activated protein kinases (MAPK) and demonstrated that this pathway naturally active in subarachnoid cysts, suggesting its involvement in the uncontrolled growth of the parasite (28). These advances provide a base framework to identify the cellular and molecular bases involved in the uncontrolled growth of the bladder wall aiming to use them as therapeutic targets to evaluate new drugs that act specifically on the germinative cells of the parasite.

Cell proliferation stimulated by insulin in the bladder wall occurs partially through the MAPK pathway (21, 28). Although insulin also exerts effects through the phosphatidyl-inositol-3-kinase (PI3K)/Akt pathway, this pathway has not yet been evaluated in the subarachnoid cyst, so their possible participation cannot be excluded.

As previously mentioned, during the processes of infection and neuroinflammation, the host responds by secreting cytokines such as TGF-β (29). This cytokine is essential for the parasite, first because it promotes the angiogenesis processes reported in parenchymal NCC favoring the access of nutrients, and second for stimulating the receptors for TGF-β present on the cyst surface stimulating the expression of essential genes for its normal development (24, 30). By analyzing the genome of some cestode species including *T. solium*, genes encoding proteins structurally similar to TGF-β have been identified (24) and could be present within the set of proteins secreted by the cyst. In fact, proteins secreted by the cyst promote Treg induction through TGF-β pathway (31, 32). The involvement of TGF-β and its signaling pathway in subarachnoid cyst development and proliferation needs to be evaluated.

WNT proteins are secreted, lipid-modified proteins that bind to receptors of Frizzled and LRP families on the cell surface (27). They play an important role in cell proliferation and parasite development by the formation of the anterior-posterior axis (27). Intracellular signaling occurs through the β-catenin protein to activate the expression of WNT target genes. Several WNT-encoded genes are expressed only in the anterior axis (scolex in *E. multilocularis*) (27), and given that the subarachnoid cyst lacks scolex, several questions remain about the possible involvement of this signaling pathway.

*T. solium*, as well as other cestodes, uptakes nutrients through the tegument of the bladder wall (33). The parasite uses glucose as the main energy source, capturing this nutrient from the host through sodium/glucose co-transporters. Previous histological studies performed on subarachnoid cyst samples reveal overexpression of the sodium/glucose co-transporter, postulating that cyst expansion occurs as a result of the passive entry of large volumes of water (34). However, the presence of mitotically active germinative cells and the tissue characteristics of the bladder wall do not support this hypothesis.

The sequencing and analysis of the *T. solium* genome have demonstrated, as in other flatworm species, the absence of genes for the *de novo* synthesis of macromolecules such as lipids (35). The parenchymal cyst supplies its demand for these nutrients by secreting fatty acid-binding proteins, thus capturing lipids from the host (36). In the case of the subarachnoid cyst, it is important to consider that the parasite develops in a special microenvironment surrounded by cerebrospinal fluid (CSF), in contrast to parenchymal cysts that are in contact with blood vessels. CSF does not provide the same nutrients or growth factors as those present in the blood circulation and the abnormal growth of the subarachnoid cyst would be associated with increased nutritional demands for glucose and lipids; however, the mechanisms or adaptations that this form of cyst employs are still unknown. The presence of iron deposits in the bladder wall of the subarachnoid cyst with increased expression of genes encoding iron-transport proteins (37) may represent an adaptation of the subarachnoid cyst to its environment to supply its energy demands associated with its continued proliferation.

The treatment for parenchymal NCC includes the use of anthelmintics (albendazole, praziquantel, or a combination of both drugs) with anti-inflammatory drugs mainly corticosteroids such as dexamethasone (19, 29). When the cysts are destroyed after the administration of anthelmintic drugs, antigens of the parasite are released, resulting in an exacerbated inflammatory response (29). This reaction may be accompanied by complications such as increased intracranial pressure and seizures. Steroids administered together with cysticidal drugs can suppress the inflammatory response and control edema that is associated with the lesions.

Subarachnoid NCC is more difficult to treat than the parenchymal form (17, 19). Long-term anti-parasitic treatment is frequently required and in many cases it fails to achieve complete parasite resolution (19). This is a consequence of the proliferative nature of the subarachnoid cyst, which can expand and invade other spaces in the CNS, also to the depletion of immune response due to long periods of corticosteroid use and to the limited capacity of the immune cells to reach the ventricles of subarachnoid space. In addition, evaluations performed on *E. multilocularis* report the limited effects of the anthelmintic drugs on germinative cells (38, 39). The identification of relevant pathways for the continued proliferation of the parasite, such as the MAPK pathway, have served to preliminarily evaluate the action of low-cost drugs targeted to this pathway for the treatment of subarachnoid NCC (28).

Subarachnoid cysts exhibit unique cellular and molecular characteristics and develop in close contact and adaptation to the host microenvironment. The use of advanced molecular techniques will allow us to understand the biology of the events that that allow its survival and trigger its aberrant growth and also to select and evaluate new therapeutic targets to improve the effectiveness of current treatment schemes while decreasing or eliminating side effects.

## Author contributions

All authors have read and agree to the published version of the manuscript. Writing-original draft preparation: MO. Review and Editing: MV, HG. All authors contributed to the article and approved the submitted version.

## Funding

This work was supported by Fogarty International Center-National Institutes of Health Training grant D43 TW001140.

## Conflict of interest

The authors declare that the research was conducted in the absence of any commercial or financial relationships that could be construed as a potential conflict of interest.

## Publisher’s note

All claims expressed in this article are solely those of the authors and do not necessarily represent those of their affiliated organizations, or those of the publisher, the editors and the reviewers. Any product that may be evaluated in this article, or claim that may be made by its manufacturer, is not guaranteed or endorsed by the publisher.
